# Analysis of clinical features of 7 cases of primary pleomorphic adenoma of lower respiratory tract and review of literature

**DOI:** 10.1097/MD.0000000000036258

**Published:** 2023-12-08

**Authors:** Ling Chen, Weidong Zhang, Xiuying Li

**Affiliations:** a Pulmonary and Critical Care Medicine, Hunan Provincial People’s Hospital, The First Affiliated Hospital of Hunan Normal University, Changsha, Hunan, China.

**Keywords:** pleomorphic adenoma, tracheal mass

## Abstract

To investigate the clinical characteristics of patients with primary pleomorphic adenoma of the lower respiratory tract. The clinical manifestations, laboratory results, pathological and imaging, treatment and prognosis of 7 patients with primary pleomorphic adenoma of the lower respiratory tract who were treated in Hunan Provincial People’s Hospital from December 2015 to May 2020 were analyzed. Among the 7 patients, 5 patients had cough and expectoration as the main clinical manifestations, and the other 2 patients had no symptoms. Pleomorphic adenomas of the lower respiratory tract are mostly located in the trachea or bronchus, and the chest computed tomography findings are circular or ellipsoid masses, or flake high-density shadows and local thickening of the tracheobronchial wall. Tumor histological features and immunohistochemistry can diagnose pleomorphic adenoma and its origin. In this study, 2 asymptomatic patients refused further treatment, 1 survived for more than 3 years, and the other was lost to follow-up during follow-up. One patient with surgical resection was followed up for 5 years after surgery and had a good survival status. The other 4 patients with respiratory symptoms who could not undergo surgery were mainly treated with bronchoscopic interventional therapy on demand, and the survival time up to now was 2 to 5 years. Primary pleomorphic adenoma of the lower respiratory tract is rare in clinic. Its clinical symptoms are related to the location and size of the tumor. Both surgical resection and bronchoscopic intervention have a good clinical prognosis. The cause of death of patients with such diseases is mostly dyspnea caused by tumors in the airway. Early diagnosis, timely intervention and regular follow-up can make patients obtain better curative effect.

## 1. Introduction

Pleomorphic adenoma is the most common histological form of salivary gland tumors such as large salivary gland, parotid gland, and submandibular gland,^[1]^ while primary lung tumors presenting as pleomorphic adenomas (PA) are extremely rare lesions and have traditionally been considered to be lesions caused by bronchial gland epithelial cells.^[2]^ We report 7 patients with primary pleomorphic adenoma, which occurred mainly in the lower respiratory tract. Most of the patients reported here had mucoepidermoid and adenoid cystic changes. The clinical features, pathology, immunohistochemistry, imaging, treatment, and prognosis of these patients were mainly analyzed, and the relevant features recorded in the previous literature were compared and analyzed.

## 2. Materials and methods

### 2.1. Case data

In this study, 7 patients diagnosed with primary pleomorphic adenoma of the lower respiratory tract according to pathological and immunohistochemical findings and without cardiopulmonary underlying diseases or other malignancies affecting survival and prognosis were selected from Hunan People’s Hospital from December 2015 to May 2020.We have obtained the patient’s informed consent form, which was approved by the Ethics Committee of Hunan Provincial People’s Hospital.

### 2.2. Diagnostic criteria

A lower respiratory tract tumor with pleomorphic adenoma was diagnosed after bronchoscopic clamp specimen or surgical excision specimen biopsy at our hospital. Histological features are composed of a mixture of 2 basic components in different proportions, namely epithelial tissue and interstitial tissue. The epithelial cells were cubic cells and columnar cells, and the epithelial components were nest-like, beamlike and glandular. The main interstitial components were oval, spindle cells, and mucoid tissue. In immunohistochemistry, cytokeratin (CK), CK7, and epithelial membrane antigen were expressed in epithelial cells, while CK, S-100 and P63 were expressed in myoepithelial cells. CKl9 was most commonly expressed in tubular glandular epithelial cells. At the molecular level, PA have 8q12 chromosome abnormalities, pleomorphic adenoma gene-1 (PLAG l) rearrangement, and high mobility group proteins A amplification.

### 2.3. Observation indicators

Observation indicators mainly included clinical features, pathological, and immunohistochemical features, imaging changes, treatment methods, and prognosis.

### 2.4. Follow-up

The main follow-up methods were outpatient review and telephone follow-up. The contents included respiratory symptoms, chest computed tomography (CT), bronchoscopy and prognosis. Follow-up time was 1, 3, and 6 months after discharge, and once a year thereafter.

## 3. Results

### 3.1. General situation and main clinical features

The main clinical features of the patients are shown in Table 1. Of the 7 patients, 4 were female and 3 were male. Ages ranged from 47 to 69 years. The tumors of 3 patients were located in the trachea, 1 patient was located in the right main bronchus, 1 patient was involved in both the left and right main bronchus, and the tumors of the remaining 2 patients were located in the peripheral bronchus. Among them, 5 patients whose lesions were located in the trachea and the left and right main bronchus had clinical symptoms, including cough, sputum, blood in the sputum, shortness of breath, and weight loss. In the other 2 patients without clinical symptoms, peripheral pulmonary lesions were found by chest CT during physical examination. Of the 7 patients, 4 were treated with bronchoscopic interventional therapy, 1 patient underwent surgical resection of the tumor. The other 2 patients were diagnosed by bronchoscopic clamp biopsy, but declined further treatment because they had no clinical symptoms.

**Table 1:** Main clinical characteristics of patients.

Case no	Sex	Age	Location/size (gross)	Clinical features	Treatment	Follow-up
1	F	59	Left lower bronchus/12mm	Cough and expectoration	Endobronchial resection	Alive and well at 2 years and 3 months
2	M	65	Left and right main bronchus/23 mm	Cough, hemoptysis, throat pain	Endobronchial resection	Died after 2 years because of recurrence of breast cancer
3	F	47	Upper lobe of left lung/Not measurable	Cough, expectoration, hemoptysis	Untreated	Lost to follow-up
4	F	49	Lower tracheal segment/6mm	Anhelation	Endobronchial resection	Alive and well at 1 years and 3 months
5	M	50	Right lower lung/18 mm	No symptoms	Lobectomy	Alive and well at 5 years and 7 months
6	M	69	Right superior lobar bronchus/not measurable	No symptoms	Untreated	Alive and well at 3 years and 1 months
7	F	59	Local thickening of tracheal wall/not measurable	Cough, expectoration, anhelation, hemoptysis	Endobronchial resection	Alive and well at 5 years

F = female, M = male.

### 3.2. Imaging manifestations

**Figure 1.**
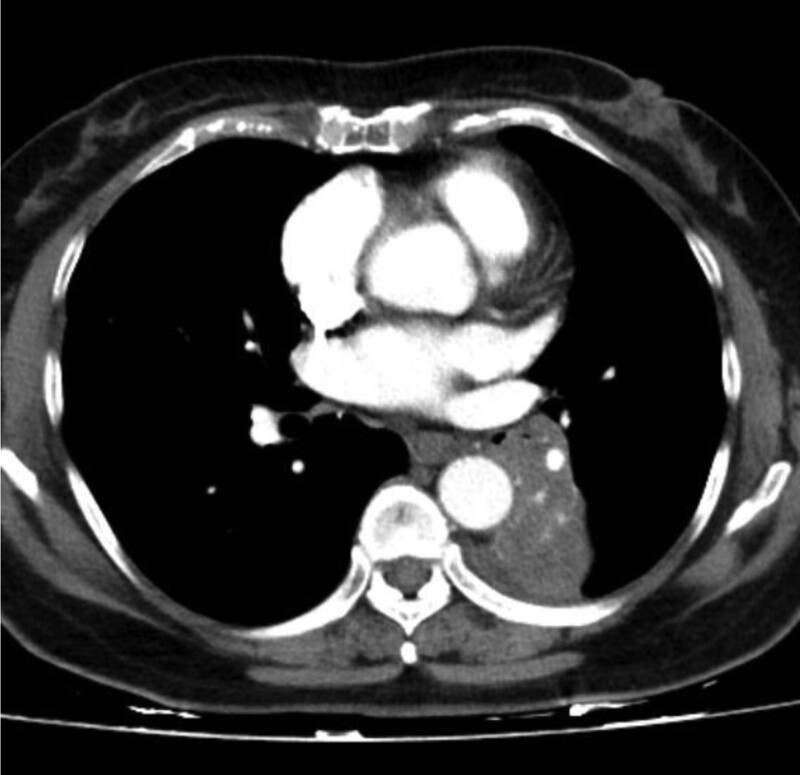
A soft tissue density lesion of about 12 mm can be seen in the bronchus of the left lower lobe of the lung, with slight enhancement at the enhancement edge.

**Figure 2.**
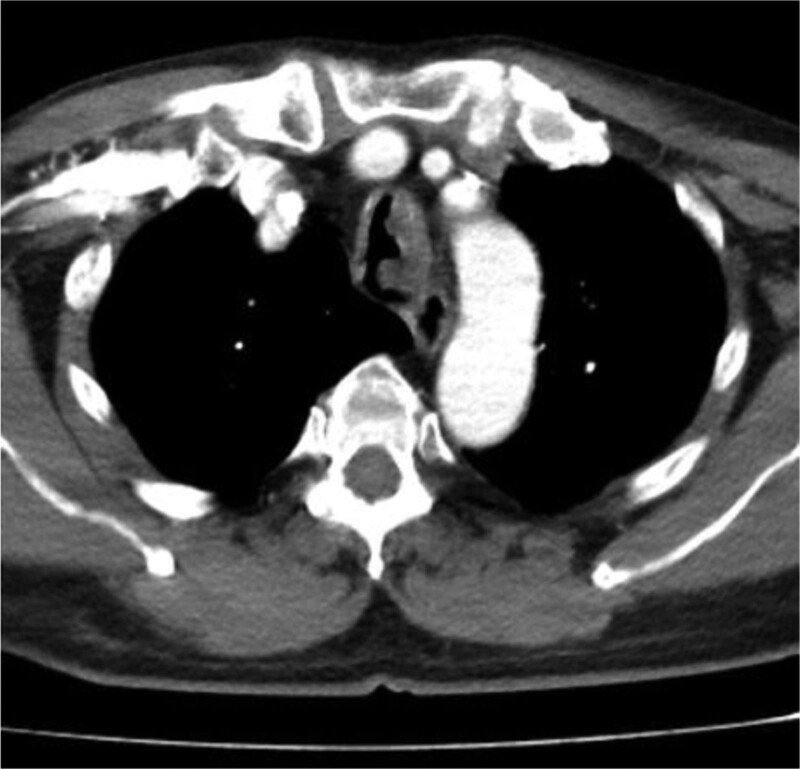
A soft tissue density lesion of 8 * 23mm can be seen in the lumen of the upper trachea, with unclear demarcation with the esophagus, and moderate enhancement.

**Figure 3.**
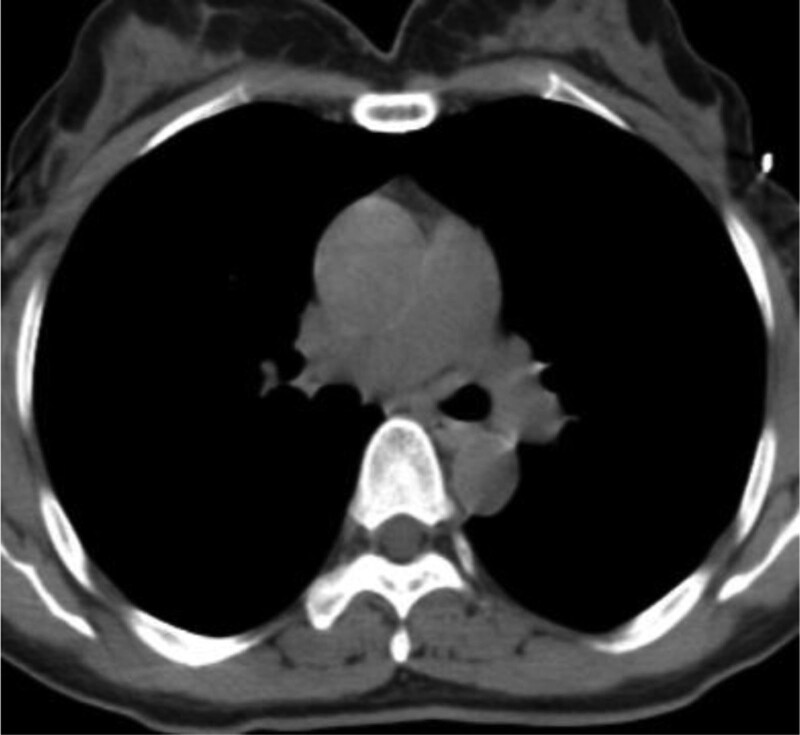
Upper lobe of left lung is speckled, patchy shadow and cystic transparent area, with blurred edge.

**Figure 4.**
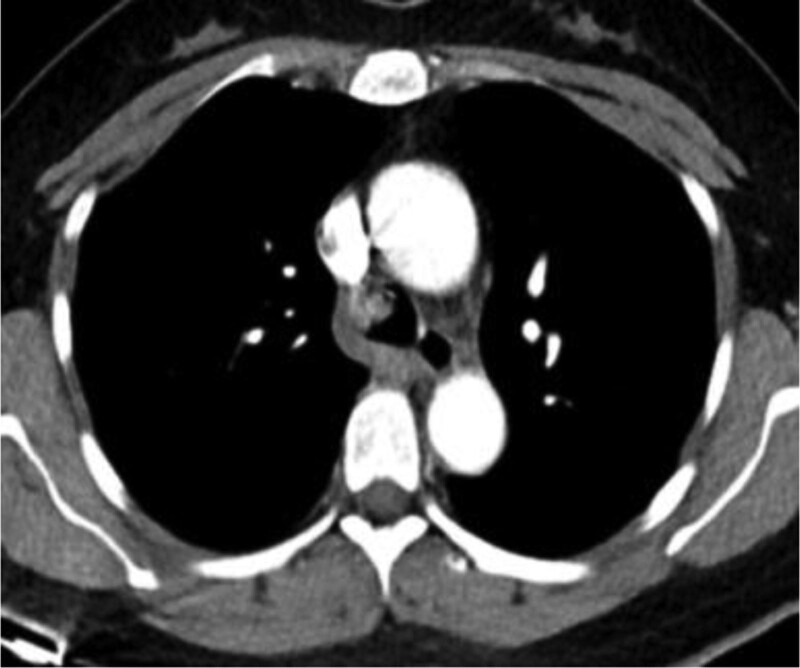
A soft tissue density nodule with a size of 6 mm can be seen on the right wall of the lower trachea, with smooth edges and slight enhancement.

**Figure 5.**
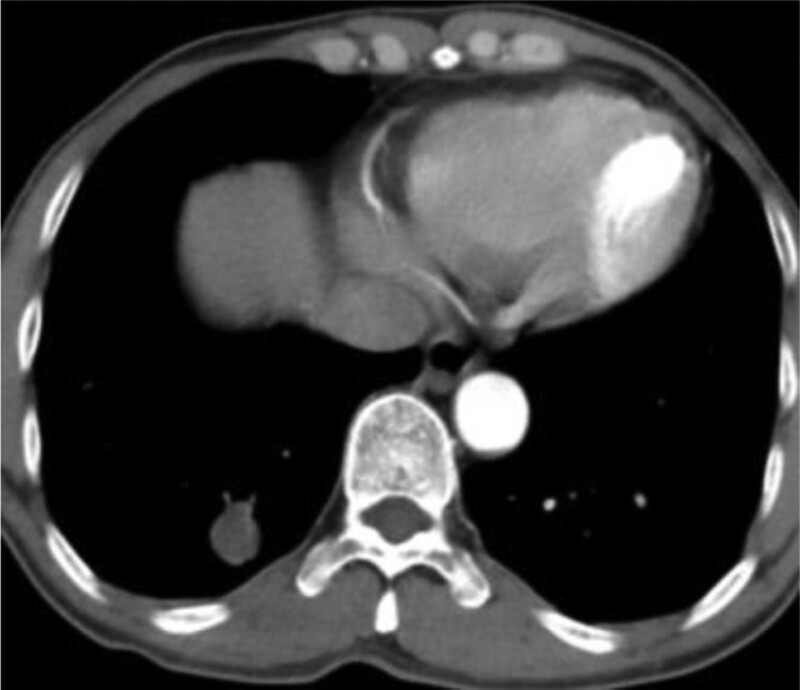
Quasi-circular nodule in the posterior basal segment of the right lower lung, about 14 mm * 11 mm in size, with clear edge, uneven density, no enhancement.

**Figure 6.**
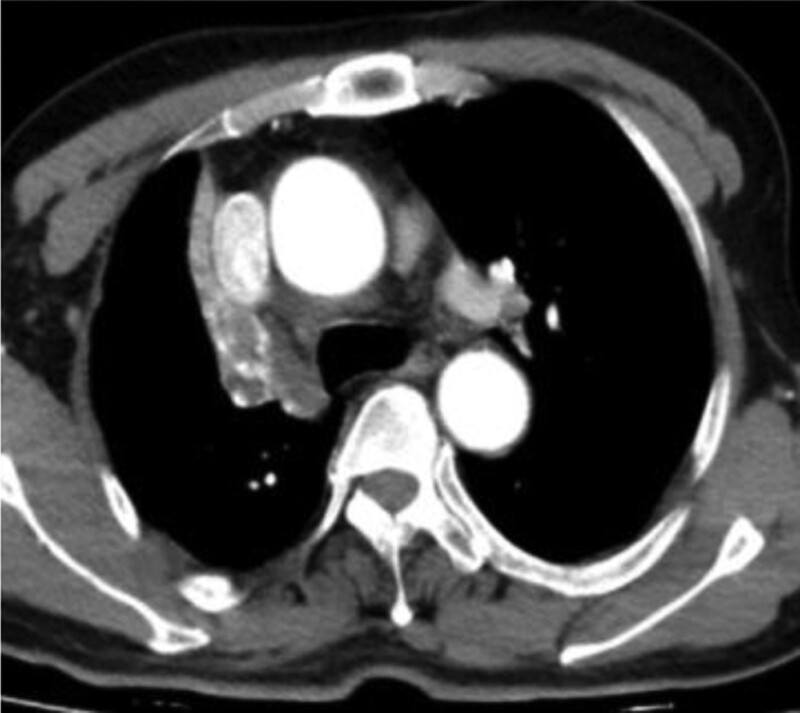
Soft tissue density foci can be seen in the right upper lung bronchus, and slight enhancement can be seen in the enhancement, and the distal right upper lung bronchus is occluded.

**Figure 7.**
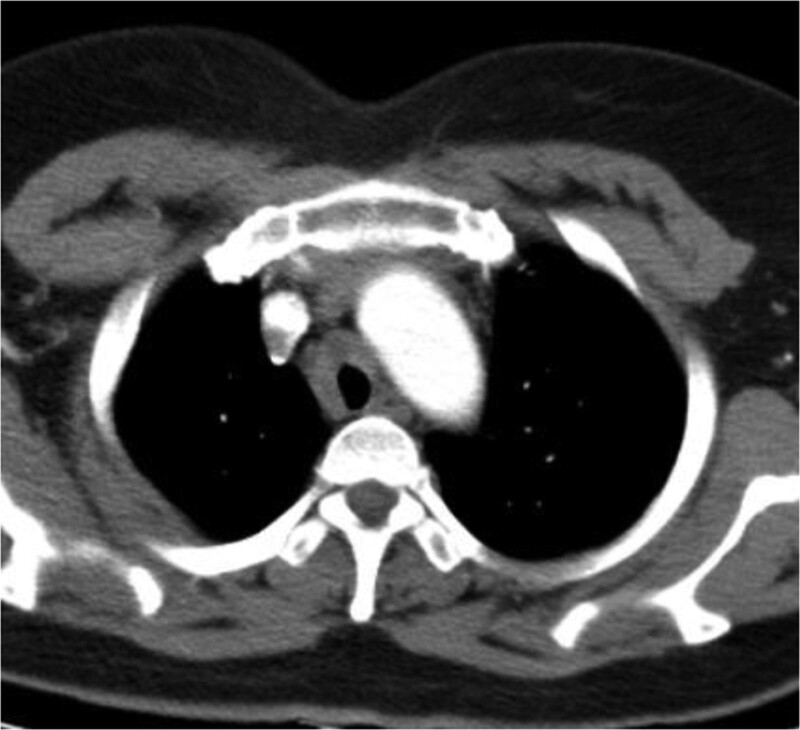
Local tracheal wall thickening, lumen stenosis, and enhancement.

In this study, lesions were found by chest CT in all 7 patients. In terms of location, shape and size of lesions, 3 patients were located in the trachea (Figs. 2, 4, and 7), 2 patients were located in the bronchus (Figs. 1 and 6), and 2 patients were located in the segmental bronchus (Figs. 3 and 5). In the cross-sectional CT images, 3 patients showed circular or ellipsoid appearance (Figs. 1, 4, and 5), 3 patients showed flake shadow (Figs. 2, 3, and 6), and 1 patient showed local thickening of trachea wall (Fig. 7). The size of the lesions in the 7 patients ranged from 8*23 mm to 5*6 mm. From the perspective of lesion edges, internal manifestations and enhancement characteristics, 4 patients had smooth lesion edges and slight enhancement after enhancement (Figs. 1, 4, 5, and 6); 3 patients had unclear boundary with surrounding tissues (Figs. 2 and 3); the other patient had local thickening of trachea wall and visible enhancement after enhancement (Fig. 7). From the perspective of lesion growth pattern and base, the lesions of 4 patients showed growth into the trachea or bronchial lumen, and the lesions were connected to the trachea or main bronchial lumen with pedicles, most of which showed broad base pattern (Figs. 1, 2, 4, and 6). From the point of view of the degree of lumen stenosis caused by the lesions: in this group, the lumen of the trachea or main bronchus presented eccentric stenosis of different degrees, and obstructive pneumonia or atelectasis was caused by lumen lesions in 2 patients (Figs. 1 and 6). In the 7 patients in this study, no pericardium or pleural effusion was found, and no enlarged lymph nodes were found in the mediastinum and hilum of the lung.

### 3.3. Treatment and outcome

Two of the 7 patients refused further treatment because they were asymptomatic. Among them, patient 3 lost follow-up after discharge, and patient 6 underwent CT examination at a local hospital 3 months and 1 year after discharge, and no abnormality was found on chest CT. Subsequently, the patient did not go back to the hospital because he was asymptomatic. Patient 5 underwent surgical resection of the right lower lung mass, and the current survival time has been 5 years and 7 months. It is worth noting that the patient underwent resection of the mixed tumor of the right lower submandibular gland in 1986, and recurred twice after surgery in 2008 and 2015, both of which were surgically resected. The other 4 patients were treated with bronchoscopic interventional therapy: Patient 1 underwent bronchoscopic argon plasma coagulation and electroentrapment. One year and 3 months after the first treatment, the patient’s tumor recurred and caused dyspnea. The symptoms were relieved after bronchoscopic resection again. At present, chest CT has not recurred, and the survival time has been 2 years and 3 months. Patient 2 underwent high-frequency electroablation and cryotherapy under bronchoscopy. Eleven months after discharge, the patient developed cough and blood in sputum. Bronchoscopy indicated severe stenosis in the middle part of the trachea, and the patient was implanted with a metal semi-coated stent under bronchoscopy. 8 months after stent implantation, the tumor recurred for the third time, and the tumor was resected under bronchoscopy. After 2 years and 3 months of survival, the patient died due to the progression of endotracheal lesions, which invaded the left and right main bronchus. After diagnosis, patient 4 was treated with bronchoscopic resection (high-frequency electric snare resection + cryoablation). There was no recurrence after surgery, and the current survival time has reached 1 year and 3 months. The symptoms of patient 7 were basically relieved after high-frequency electric and argon knife ablation under bronchoscopy. However, tumor recurred 1 year later, requiring bronchoscopic interventional therapy every 3 to 5 months. At the third year after diagnosis, the patient underwent bronchoscopic stent implantation in another hospital. At present, bronchoscopic interventional therapy is required to clear the airway every 1 to 2 months to relieve dyspnea, and the overall survival time has been as long as 5 years.

### 3.4. Pathological results and immunohistochemistry

**Figure 8.**
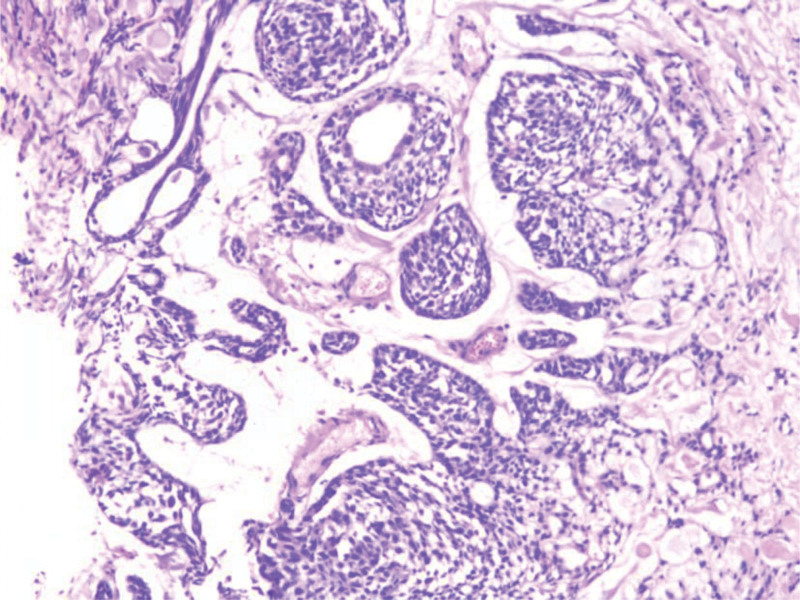
Pleomorphic adenoma: The tumor cells are arranged in a complex structure, forming nests and double glandular tubes, which are composed of inner glandular epithelium and outer myoepithelium. The tumor cells have mild atypia and pathological nuclear division is rare (HE, ×20).

**Figure 9.**
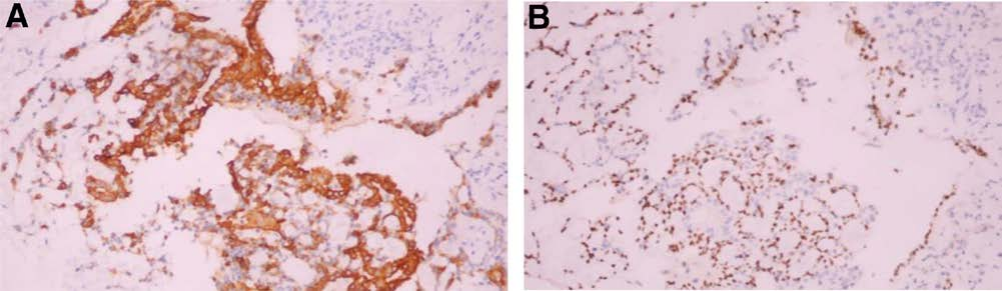
Immunohistochemical staining results of tumor (A) the expression of CK7 was positive in the glandular epithelial cells (Envision, ×20) (B) the expression of P63 was positive in the myoepithelial cells (Envision,×20).

The histological features and immunohistochemistry of the tumors in the 7 patients were considered to be tumors of salivary gland origin, with incomplete or no envelope, showing similar features to tumors in the salivary gland, and were composed of 2 basic components, namely epithelial tissue and interstitial tissue. Among them, necrosis and calcification were observed in the tumor of patient 1, and no cartilage tissue was observed in all the 7 patients. The epithelial components were nest-like, beamlike and glandular, and the epithelial cells were cubic and columnar. Most patients showed a mixed composition of myoepithelium and glandular epithelium. The interstitial components were oval, spindle cells and mucoid tissue, among which the tumor cells of patient 7 had certain atypia, and it was considered to be borderline malignant. The pathological samples of all patients were taken and made, and the immunohistochemical results were improved to determine the tissue source, and all 7 patients showed p63 (+). 5 patients had CK7 (+), CK19 (+), CK5/6 (+); all patients were positive for Ki67 (< 10% in 4 cases and 30% in 3 cases). Four patients had smooth muscle antibody (+) and Syn (−) (Figs. 8 and 9).

## 4. Discussion

Lung parenchymal tumors are the main tumors in the respiratory system, while trachea tumors are not common. An American study^[3]^ showed that primary tumors of trachea only accounted for 2% of malignant tumors of the respiratory system. However, primary tumors of the trachea include adenoid cystic carcinoma, mucoepidermoid carcinoma and pleomorphic adenoma, among which pleomorphic adenoma only accounts for about 2%.^[4]^ Pleomorphic adenoma often occurs in salivary gland tissue,^[5]^ while it rarely occurs in the lower respiratory tract.^[6]^ Therefore, the previous literature on pleomorphic adenoma of trachea is mostly about case reports. According to some research statistics,^[7,8]^ as of 2011, only more than 50 cases of pleomorphic adenoma of lung trachea and bronchus have been reported in foreign literature, while <10 cases have been reported in China. In recent years, the number of domestic reports has gradually increased. At the time of writing this article, the domestic and foreign literature reports retrieved from CNKI, Wanfang, PUBMED, Metrotron, and other databases are mostly individual reports.

### 4.1. Clinical features of PA

In general, tracheal PA show no difference in gender distribution, but there are differences in age distribution. In the study of Qian-Nuan et al,^[9]^ it was pointed out that tracheal PA tend to occur in middle age, with an average age of 48 years old, which is consistent with the age at which tracheal PA occur in this study. However, there are also reports of special cases. For example, Baghai-Wadji M et al^[10]^ reported an 8-year-old child suffering from pleomorphic adenoma of trachea. The clinical symptoms of pleomorphic adenoma are often related to the size and location of the tumor and the relationship between the tumor and bronchus and surrounding tissues.^[11]^ The lesions often involve the trachea and may be asymptomatic and asymptomatic in the early stage. When the mass gradually increases to block more than 1/2 of the airway lumen, the patients often show symptoms of obstruction, such as cough, sputum, gasping, and wheezing. At this time, coherent inspiratory rales, and even bronchial obstruction signs such as seated breathing and triscopal sign of inspiratory phase may appear, so it is easily misdiagnosed as bronchial asthma in clinical practice.^[12,13]^ In this study, 5 patients had clinical symptoms, mainly manifested as shortness of breath, cough, and expectoration, among which 3 patients had blood in the sputum, possibly due to tumor invasion of blood vessels. On the other hand, the lesion may be asymptomatic when it is located in the lung parenchyma or distal bronchus, and is often detected during physical examination.^[14,15]^ At present, low-dose spiral CT is widely used in physical examination, and the detection rate of airway tumors is getting higher and higher. In this study, 2 patients were found during physical examination.

### 4.2. Imaging manifestations of tracheal pleomorphic adenoma

Bronchial PA are often missed on chest radiographs, and most patients are found on chest CT. High-resolution CT has obvious advantages in the detection of tumors in trachea and bronchus, and all patients in this study were found on CT. CT can clearly show the location, size, shape, degree of lumen stenosis, and whether the base is connected to the tube wall. Pleomorphic adenomas occurring in the lower respiratory tract may be located in the trachea and bronchus, as well as in the lung parenchyma or mediastinum.^[15]^ Literature has concluded that nearly half of PA of the trachea are located in the upper third of the trachea, and 12% are located in the lower third.^[16]^ The tumors vary in diameter and shape. Liao, Chen Ming et al^[15,17]^ agreed in the case summary that the size of pleomorphic adenoma of trachea is mostly about 2 cm, which is consistent with the tumor size of the 7 patients in this study. Most of the individual cases of tracheal PA have been reported as intratracheal polyps, and some of them may be pulmonary nodules or lamellar shadows.^[18,19]^ Most of the patients in this study showed circular or oval nodules with clear internal boundaries of trachea and bronchus. Particularly, patient No. 7 only showed thickening of the airway wall on CT, but no obvious nodules, which may be related to the early stage of the disease. In their study, Chen Ming et al^[15]^ summarized the imaging features of pleomorphic adenoma that should be considered clinically: CT showed that the luminal growth of the main bronchus of the trachea was of regular shape and more uniform density, and the enhanced scan showed a “slow in and slow out” type of obvious enhancement, accompanied by different degrees of stenosis of the trachea or main bronchus lumen, without mediastinum and hilar lymph node enlargement and pleural effusion. In addition, positron emission tomography CT can also provide some information for malignant component analysis and true tumor size of tracheal pleomorphic adenoma, and is of certain value for long-term follow-up of patients.^[20]^

### 4.3. Treatment and prognosis of tracheal pleomorphic adenoma

At present, the number of reported cases of endotracheal pleomorphic adenoma is small, so the best treatment and prognosis of this disease are still unclear, but the main therapeutic principles are complete resection of the tumor, prevention of recurrence and relief of airway obstruction.^[21]^ The main treatment methods for tracheal pleomorphic adenoma include surgical resection and bronchoscopic tumor resection.^[22,23]^ Most surgical procedures are lobectomy in cardiothoracic surgery,^[16,21,23–25]^ and some patients are treated in otorhinolaryngology surgery by tumor resection after tracheotomy.^[26]^ In this study, patient No. 5 was selected for surgical resection, which was a wedge resection of the right lower pulmonary nodules of video-assisted thoracic surgery under general anesthesia. The patient had a good survival status 5 years and 7 months after surgery. Bronchoscopic intervention is another important treatment method.^[27,28]^ In previous literatures, the methods of bronchoscopic intervention are also different, including flexible bronchoscopic high-frequency electric snare + electrocoagulation, argon ion beam coagulation, thermal ablation therapy such as laser, and intraairway interventional therapy such as cryotherapy.^[29–31]^ In this study, 4 patients were selected for endobronchial interventional therapy, and 2 of them were given endobronchial stent placement, which may be related to the fact that this study mainly included respiratory patients, but these 4 patients showed good curative effects after treatment. Endoscopic resection of tumors provides treatment opportunities for patients with poor cardiopulmonary function who cannot tolerate surgery, and avoids lung function damage caused by surgery. In this study, although patient No. 6 refused treatment, the current survival time was more than 3 years and 1 month, which shows that the growth of tracheal pleomorphic adenoma is relatively slow in most cases and the prognosis is good. However, some tracheal PA metastasized or recurred after surgery, and Moran CA et al pointed out that the prognosis of patients may be related to tumor size, degree of local invasion, and degree of tumor division activity.^[32]^ In this study, 3 patients had tumor recurrence after resection, requiring repeated tracheoscopic tumor resection, which was consistent with the conclusion mentioned in most studies that the tumor had low malignant potential,^[33–35]^ which required us to inform patients that regular review and follow-up should be conducted after tumor resection.

### 4.4. Pathology and immunohistochemistry of tracheal pleomorphic adenoma

Tracheal pleomorphic adenoma, also known as mixed tumor, is characterized by the diversity of cell morphology and structure, with epithelial and mesenchymal tissue composition as its basic diagnostic features. There are many types of epithelial cells, including fusiform, clear, squamous, basal-like, eosinophilic, mucous, and sebaceous cells, which can be transformed from cell to cell. These cells are often atypical and rarely have nuclear division. Mesenchymal components are divided into mucoid and hyalomatous tissues, which accumulate in or around epithelial cells. Unlike PA occurring in salivary glands and other organs, it generally does not have cartilage tissue, and in some cases, squamous metaplasia can be seen.^[36]^ Immunohistochemistry is used to determine the tissue origin of PA. In general, the expression of ductal and myoepithelial cells is: Low molecular weight CK (CAM2.5), broad spectrum CK, Thyroid transcription factor 1, PE-10, myoepithelial cells, and stromal cells expressed Vimentin, smooth muscle antibody, glial fibrillary acidic protein, epithelial cells expressed CK, epithelial membrane antigen, CK7, myoepithelial cells often expressed CK, P63 and S-100. CKl9 and CK7 were most commonly expressed in tubular glandular epithelial cells. Most patients showed a mixture of myoepithelial and glandular epithelium. Positive Ki67 indicates the proliferation rate of tumor cells,^[37]^ which is <3% for normal cells, about 5% to 10% for benign tumors, and usually >10% for malignant tumors. In this study, 2 patients showed a low proliferation rate and 3 patients showed a high proliferation rate. Sometimes PA resemble aggressive epithelial-origin tumors and are misdiagnosed as carcinomas because they are rich in epithelial cell components and lack interstitial components.

In conclusion, primary PA of the lower respiratory tract are rare in clinical practice, and their clinical symptoms are related to the location and size of the tumor. Both surgical resection and interventional bronchoscopic treatment have a good clinical prognosis. The cause of death of patients with such diseases is mostly dyspnea caused by tumors in the airway. Early diagnosis and timely intervention can make patients obtain better curative effect. As this disease is relatively rare in clinic, the sample size of this study is small and the patients enrolled in the respiratory department are mainly bronchial or endobronchial primary pleomorphic adenoma, which may have a certain influence on the choice of treatment. Due to the low incidence of this disease, there is currently no guideline or consensus to standardize the diagnosis and treatment plan of this disease. Although 7 patients were included in this study for observation study, it is still necessary to further explore the best treatment and efficacy of this disease in a multicenter, large-sample study, so as to enhance the universality and applicability of the research results and improve the external validity.

## Author contributions

**Investigation:** Weidong Zhang.

**Project administration:** Xiuying Li.

**Resources:** Xiuying Li.

**Supervision:** Weidong Zhang.

**Validation:** Weidong Zhang.

**Writing – original draft:** Ling Chen.

**Writing – review & editing:** Ling Chen.

## References

[1] Cui JT Ni YQ Li C. Pleomorphic adenoma of trachea: a case report. J Intractable Dis. 2020;19:191–2.

[2] Kim BG Lee K Um SW. Clinical outcomes and the role of bronchoscopic intervention in patients with primary pulmonary salivary gland-type tumors. Lung Cancer. 2020;146:58–65. 32512274 10.1016/j.lungcan.2020.05.016

[3] Young JL Percy CL Asire AJ. Cancer incidence and mortality in the United States, 1973-77. Natl Cancer Inst Monogr. 1981;57:1–187. 7278952

[4] Gaissert HA Mark EJ. Tracheobronchial gland tumors. Cancer Control. 1999;13:286–94. 10.1177/107327480601300406 17075566

[5] Almeslet AS. Pleomorphic adenoma: a systematic review. Int J Clin Pediatr Dent. 2020;13:284–7. 32904077 10.5005/jp-journals-10005-1776 PMC7450192

[6] Falk N Weissferdt A Kalhor N. Primary pulmonary salivary gland-type tumors: a review and update. Adv Anat Pathol. 2016;23:13–23. 26645458 10.1097/PAP.0000000000000099

[7] David AP Bakos SR Daniero JJ. Pleomorphic adenoma of the trachea. Ear Nose Throat J. 2020;99:235–6. 30987459 10.1177/0145561319840189

[8] Takahashi M Yorozuya T Miyasaka Y. A case of tracheal pleomorphic adenoma misdiagnosed as asthma. Oxf Med Case Reports. 2019;2019:omz111. 31777662 10.1093/omcr/omz111 PMC6874862

[9] Liao Q-N Fang ZK Chen SB. Pleomorphic adenoma of the trachea: a case report and review of the literature. World J Clin Cases. 2020;8:198–207. 10.12998/wjcc.v8.i23.6026 PMC7723722 33344601

[10] Baghai-Wadji M Sianati M Nikpour H. Pleomorphic adenoma of the trachea in an 8-year-old boy: a case report. J Pediatr Surg. 2006;41:e23–6. 10.1016/j.jpedsurg.2006.04.008 16863832

[11] Li D Wang WJ An QX. Diagnosis and treatment analysis of a case of bronchial pleomorphic adenoma and literature review. China Gen Med. 2018;03:111–6.

[12] Fitchett J Luckraz H Gibbs A. A rare case of primary pleomorphic adenoma in main bronchus. Ann Thorac Surg. 2008;86:1025–6. 18721613 10.1016/j.athoracsur.2008.02.073

[13] Von Stecher F Lewkowicz MLM Bruno A. [Unexpected cause of tracheal obstruction: pleomorphic adenoma with lipometaplasia]. Medicina. 2022;82:138–41. 35037872

[14] Ali SR Arrossi AV Mehta AC. Endobronchial pleomorphic adenoma. Oxf Med Case Reports. 2016;2016:omw090. 28031854 10.1093/omcr/omw090 PMC5184838

[15] Chen M Wang YY Jiang YH. Preliminary study on CT manifestations of pleomorphic adenoma of the tracheal main bronchus. J Clin Radiol. 2019;38:196–9.

[16] Aribas OK Kanat F Avunduk MC. Pleomorphic adenoma of the trachea mimicking bronchial asthma: report of a case. Surg Today. 2007;37:493–5. 17522768 10.1007/s00595-006-3441-0

[17] Liao QN Fang ZK Chen SB. Pleomorphic adenoma of the trachea: A case report and review of the literature. World J Clin Cases. 2020;8:6026–35. 33344601 10.12998/wjcc.v8.i23.6026 PMC7723722

[18] Motoi N Ishikawa Y. Salivary gland-type neoplasm of the lung. Diagnostic Histopathol. 2014;20:398–404.

[19] Baghai-Wadji M Sianati M Nikpour H. Pleomorphic adenoma of the trachea in an 8-year-old boy: a case report. J Pediatr Surg. 2006;41:e23–6. 10.1016/j.jpedsurg.2006.04.008 16863832

[20] Yang CH Liu NT Huang TW. Role of positron emission tomography in primary carcinoma ex pleomorphic adenoma of the bronchus: a case report. World J Clin Cases. 2021;9:2811–5. 33969063 10.12998/wjcc.v9.i12.2811 PMC8058676

[21] Leng ZJ Xia HL Cao LJ. Tracheal pleomorphic adenoma and painless bronchoscopy interventional therapy. J Clin Pulmonol. 2015;(09):195–7.

[22] Yang HM Yin J Li G. Clinical features and interventional bronchoscopic treatment of primary airway tumor in 8 children. Zhonghua Er Ke Za Zhi. 2021;59:27–32. 33397000 10.3760/cma.j.cn112140-20200904-00849

[23] Liu YG Zhao H Hua XM. Experience of surgical treatment of tracheal pleomorphic adenoma. China Pract Med. 2011;06:103–5.

[24] Ding C Yap WM Teo C. Tracheal carcinoma ex pleomorphic adenoma: a rare tumour with potential problems in diagnosis. Histopathology. 2010;51:868–71. 10.1111/j.1365-2559.2007.02865.x 17916072

[25] Karamustafaoglu YA Yanik F Yoruk Y. Palliative treatment of recurrent tracheal pleomorphic adenoma 10 years after segmental resection using the endobronchial shaver. Clin Respir J. 2020;14:495–7. 31916406 10.1111/crj.13149

[26] Zhang QQ Song XC Zhang H. Tracheotomy for two cases of tracheal pleomorphic adenoma. Chinese J Otolaryngol Head Neck Surg. 2009;44:1039–40. 20193623

[27] Matsubara M Yasuo M Tanabe T. Pleomorphic adenoma with an endobronchial resection. Intern Med. 2008;47:1117–20. 18552469 10.2169/internalmedicine.47.0853

[28] Kajikawa S Oki M Saka H. Pleomorphic adenoma of the trachea. Respiration. 2010;80:433–4. 20357424 10.1159/000308462

[29] Juarez MM Albertson TE Chan AL. Interventional bronchoscopy for obstructing benign airway tumors: which modality is ideal. J Thorac Dis. 2011;3:217–8. 22263094 10.3978/j.issn.2072-1439.2011.06.03 PMC3256535

[30] Miller S Bellinger C Chatterjee A. Argon plasma coagulation and electrosurgery for benign endobronchial tumors. J Bronchol Intervent Pulmonol. 2013;20:38–40. 10.1097/LBR.0b013e318282d3ca 23328141

[31] Guo JQ Chen ZX Tu HY. Clinical analysis of 26 cases of benign airway tumors treated by fiberoptic bronchoscopy with laser. Chinese J Endosc. 2005;11:19–21.

[32] Moran CA Suster S Askin FB. – Benign and malignant salivary gland-type mixed tumors of the lung Clinicopathologic and immnohistocemical study of eight cases. Cancer. 1994;73:2481–90. 7513602 10.1002/1097-0142(19940515)73:10<2481::aid-cncr2820731006>3.0.co;2-a

[33] Gakidis I Mihos PT Chatziantoniou C. A large neglected pleomorphic adenoma of the lung: report of a rare case. Asian Cardiovasc Thoracic Annals. 2014;22:620–2. 10.1177/0218492313479959 24867040

[34] Sakamoto H Uda H Tanaka T. Pleomorphic adenoma in the periphery of the lung. Report of a case and review of the literature. Arch Pathol Lab Med. 1991;115:393–6. 2012502

[35] Inomata M Kuroki S Oguri N. Pleomorphic adenoma of the trachea: a case report. Int J Surg Case Rep. 2023;109:108499. 37459695 10.1016/j.ijscr.2023.108499 PMC10439302

[36] Zheng HH Zhou MY Weng SX. Clinicopathological features of primary salivary gland tumors of the lung. Zhejiang Pract Med. 2021;(26):234–47.

[37] Díaz KP Gondak R Martins LL. Fatty acid synthase and Ki-67 immunoexpression can be useful for the identification of malignant component in carcinoma ex-pleomorphic adenoma. J Oral Pathol Med. 2019;48:232–8. 30597641 10.1111/jop.12820

